# The health equity measurement framework: a comprehensive model to measure social inequities in health

**DOI:** 10.1186/s12939-019-0935-0

**Published:** 2019-02-19

**Authors:** Douglas C. Dover, Ana Paula Belon

**Affiliations:** 1 Alberta Health, Government of Alberta, Edmonton, AB Canada; 2 Concordia University of Edmonton, Edmonton, AB Canada; 3 School of Public Health, University of Alberta, Edmonton, AB Canada

**Keywords:** Health equity, Inequities, Healthcare, Social determinants of health, Public health surveillance, Framework

## Abstract

**Background:**

Despite the wealth of frameworks on social determinants of health (SDOH), two current limitations include the relative superficial description of factors affecting health and a lack of focus on measuring health equity. The Health Equity Measurement Framework (HEMF) addresses these gaps by providing a more encompassing view of the multitude of SDOH and drivers of health service utilisation and by guiding quantitative analysis for public health surveillance and policy development. The objective of this paper is to present the HEMF, which was specifically designed to measure the direct and indirect effects of SDOH to support improved statistical modelling and measurement of health equity.

**Methods:**

Based on a framework synthesis, the HEMF development involved initially integrating theoretical components from existing SDOH and health system utilisation frameworks. To further develop the framework, relevant publications on SDOH and health equity were identified through a literature review in major electronic databases. White and grey literatures were critically reviewed to identify strengths and gaps in the existing frameworks in order to inform the development of a unique health equity measurement framework. Finally, over a two-year period of consultation, scholars, health practitioners, and local policy influencers from municipal and provincial governments provided critical feedback on the framework regarding its components and causal relationships.

**Results:**

This unified framework includes the socioeconomic, cultural, and political context, health policy context, social stratification, social location, material and social circumstances, environment, biological factors, health-related behaviours and beliefs, stress, quality of care, and healthcare utilisation. Alongside the HEMF’s self-exploratory diagram showing the causal pathways in-depth, a number of examples are provided to illustrate the framework’s usefulness in measuring and monitoring health equity as well as informing policy-making.

**Conclusions:**

The HEMF highlights intervention areas to be influenced by strategic public policy for any organisation whose purview has an effect on health, including helping non-health sectors (such as education and labour) to better understand how their policies influence population health and perceive their role in health equity promotion. The HEMF recognises the complexity surrounding the SDOH and provides a clear, overarching direction for empirical work on health equity.

**Electronic supplementary material:**

The online version of this article (10.1186/s12939-019-0935-0) contains supplementary material, which is available to authorized users.

## Background

Health equity and the social determinants of health (SDOH) are at the forefront of contemporary public health. As health inequities are the product of social injustice and are avoidable [1], a focus on the upstream SDOH is needed to improve population health and promote health equity. Public health and other sectors have worked collaboratively to integrate the health equity agenda into programs and policies [2]. Measuring health equity is a critical step to promote opportunities for all people - regardless of their social background - to live healthily and longer and to monitor progress in health and intersectoral strategies [3]. Identifying where to intervene effectively to promote health equity has to come from an acknowledgement of the underlying complex causal structure. This implies that measuring the interrelationships between different SDOH and health is a pivotal component to understanding and acting on health equity.

Public health has addressed this issue primarily through the use of frameworks. A wealth of guiding frameworks describing the wide variety of social mechanisms affecting health are available (e.g., Determinants of Health Model [4] and WHO’s Commission on Social Determinants of Health conceptual framework [5]). However, the two gaps in the existing frameworks are the lack of depth identifying SDOH and the lack of focus on measuring health equity. For these frameworks to be useful in health equity surveillance, they must be amenable to measurement [6]. Some frameworks have incorporated causal pathways between SDOH and health with statistical models. For instance, Ansari et al. [3] presents a causal model capturing the broad categories of SDOH, health care system attributes, (un)healthy behaviours, and health outcomes with the objective of testing pathways. Diderichsen et al.’s [7] model also explicitly incorporates causal pathways of differential exposure, vulnerability and consequences. However, these and similar frameworks lack details on the SDOH influencing health equity.

We propose an expanded, more descriptive framework to better capture the complexity of SDOH on the generation of health (in)equity. Called the Health Equity Measurement Framework (HEMF), it is designed to describe the multitude of SDOH in a causal framework and guide the quantitative analysis of health equity for ongoing public health surveillance and policy development. The HEMF has several advantages over the current SDOH-related frameworks. First, its rich visual depiction of the multiple factors affecting health offers a unified theoretical framework on social determinants of both health and of healthcare utilisation for testing hypotheses. Second, the HEMF presents a self-exploratory graphic scheme and an accompanying detailed conceptual guide that facilitate its use by multiple stakeholders including governments, non-government agencies, students, academics, and health practitioners. Third, it accommodates cross-sectional and longitudinal analyses (including life-course approaches). Finally, the HEMF is useful and practical in applied public health research, epidemiology, and public health surveillance. Our article aims to introduce the HEMF and present the theoretical components of other well-known frameworks that informed its development. Examples are also provided suggesting future uses of the HEMF in guiding health equity measurement and policy-making.

## Methods

The process of developing the HEMF was based on a framework synthesis, which involved integrating existing frameworks, narrative literature review, and consultation with potential knowledge users. The cornerstone of the HEMF is the well-known WHO’s Commission on Social Determinants of Health conceptual framework [5]. Based upon Diderichsen et al.’s [7] health equity model illustrating the causal pathways, WHO’s framework includes structural determinants of health inequities and intermediary determinants of health. Its main purpose is to drive action in reducing health inequities. To complement the WHO’s framework, we drew on the Alberta Quality Matrix for Health (AQMH) developed by Health Quality Council of Alberta [8] to include aspects related to health services use. The AQMH is a conceptual framework divided into two components: 1) six dimensions of health service quality and 2) four areas of health needs of patients and public. The behavioural model of health services utilisation produced by Aday and Andersen [9] was used to guide the interrelationships in access and equity in healthcare utilisation. These frameworks were synthesised from the perspective presented in Ansari et al.‘s [3] public health model of SDOH which allows for measuring and testing the factors linking SDOH, healthcare system attributes, health behaviours, and health outcomes.

Thereafter, we aimed to identify different components described by other SDOH-related models that may not have been included or fully described in the WHO’s SDOH or AQMH frameworks. In order to integrate key components in our unique framework dedicated to health equity measurement, we used the recent comprehensive reviews of SDOH by Mikkonen and Raphael [10] and SDOH frameworks by the Canadian Council on Social Determinants of Health (CCSDH) [11]. The CCSDH’s review also helped examining the strengths and gaps of the existing frameworks. Mutually exclusiveness and exhaustiveness were the parameters that guided our work when combining components of WHO’s SDOH, AQMH, and other frameworks. Therefore, the HEMF components are agglomerations of factors that share similar mechanisms affecting health or other constructors.

Once the HEMF components were defined, we conducted a narrative literature review to identify theoretical and empirical work from medicine, epidemiology, health promotion, and social sciences on SDOH, health equity, and social inequalities in health. This non-exhaustive literature review was meant to help to conceptualise each HEMF component and identify empirical evidence to support the definition of the casual pathways. To identify relevant, peer-reviewed publications, we performed electronic searches in major scientific databases: MEDLINE/Ovid, Sociological Abstracts/ProQuest, Web of Science/Clarivate Analytics, and Google Scholar. Examples of search terms included SDOH, social inequalities, social inequities, social disparities, and health differences. We also searched publications from well-known experts in the field of SDOH, health equity, and specific components (e.g., social capital, social cohesion, and social location). Grey literature describing and summarising frameworks was also examined.

To better connect the components from a health equity measurement perspective, we recruited a convenience sampling of potential knowledge users of the HEMF from a variety of fields within academic and governmental settings. At the stages of conceptualisation of the framework components and definition of causal relationships, they were invited to critically reflect on the causal pathways, completeness, and applicability of the HEMF in their work.

This participatory and iterative approach occurred over a two-year period and involved consultations at professional and academic meetings with approximately: a) 30 staff (from managers to analysts to embedded researchers) of a provincial ministry of health and health region departments of surveillance, performance measurement, health promotion, health prevention, health research, health services research, and policy development; b) 20 health surveillance professionals across Canada ranging from directors to local health region practitioners; c) 10 academics in Canada from the areas of epidemiology, social sciences, health services research, and biostatistics; d) 10 medical officers of health at both the provincial and federal levels; e) 10 stakeholders from local community organisations, a local municipality, and provincial ministries responsible for social services and labour. The elicitation of feedback helped refine the HEMF by addressing gaps in components or in the relationships between them, validating and clarifying concepts, and ensuring the potential for application in the area of equity surveillance and measurement.

**Fig. 1:**
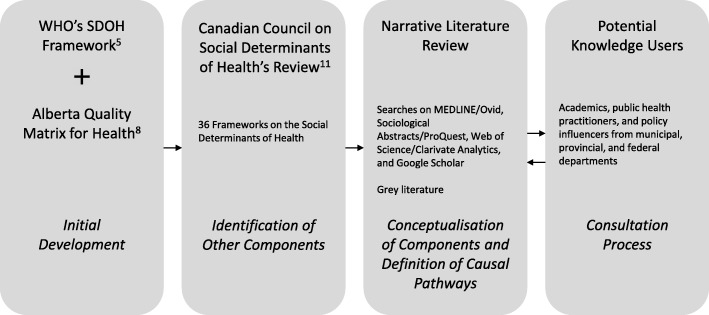
Development Process of the Health Equity Measurement Framework

To summarise, the HEMF is a synthesis of theoretical components from existing SDOH and health system utilisation frameworks and current literature, as well as reflections from potential knowledge users in academia and government (Fig. 1). Additional file 1 contains an overview of these key frameworks, the perspectives they provide, and additional key concepts contributing to the development of the HEMF.

## Results

### The HEMF components

**Fig. 2:**
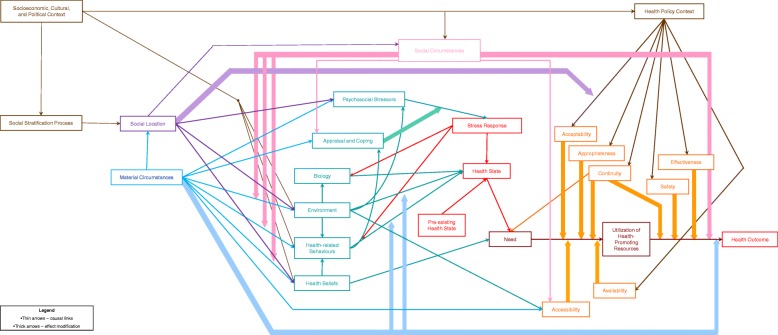
The blue line between "Social Location" and "Material Circumstances" should be double headed, having an arrow head at both ends

Figure 2 shows the HEMF. Each concept (box in the figure) is now described along with its causal relationships (thin arrows) and its effect modifications (thick arrows).

### Socioeconomic, cultural and political context

Socioeconomic, Cultural and Political Context refers to the structure of the society and the socioeconomic, political, cultural, and functional mechanisms through which it operates [5]. It includes government apparatus, political traditions, financial institutions, transnational corporations, labour markets, citizens’ legal rights and obligations, and sociocultural values and norms, etc. The socioeconomic and political contexts determines the availability, distribution, and quality of public infrastructure and resources, like housing, education, social security, work regulations, and healthcare [12], whereas the sociocultural context shapes the nature of social relationships and values attributed to health.

Partially because there is no direct measure to capture the contextual influence at the individual level [5], the broad context has been less investigated and explicitly recognised in health studies [12–14]. However, as a powerful determinant in the formation and reproduction of social structure and a driving force in policy development and implementation, the Socioeconomic, Cultural and Political Context has an impact on social distribution of health and people’s opportunities to be healthy [5, 13] .

As seen in the framework, this context influences the Social Stratification Process, Health Policy Context, Environment, Health-related Behaviours and Health Beliefs, and Social Circumstances. The context creates social stratification mechanisms, which are context- and time-specific. Some policies may generate segregation, exclusion, and discrimination, while others may mitigate social inequities (e.g., public vaccination campaigns or conditional cash-transfer programs) [5].

The public social values attributed to health (individual/private or collective concern [5]) influence the extent to which the government addresses healthcare systems and health policies. Context determines the eligibility criteria and coverage of public health insurance, provision and quality of medical services and treatments (including their Acceptability, Appropriateness, Continuity, Availability, Effectiveness, and Safety), offers of immunisation programs, development of clinical guidelines, regulation of private healthcare sector, etc. Also, the broader Context shapes both natural and built Environment, affecting, for instance, people’s access to good air quality, mixed-use neighbourhoods, and affordable public transportation.

The Context shapes patterns of Health-Related Behaviours and Beliefs [1]. For instance, cultural norms associating cigarette smoking to adulthood and independence play a role in the high prevalence of adolescent smokers. Healthy public policies may increase awareness of health benefits associated with flu shots and nutritious diet, leading to adoption and maintenance of healthy behaviours. However, the omission or ineffectiveness of some policies may lead to increased prevalence of health-damaging behaviours, such as drug addiction. Also, culture, norms, and values in the Context shape individual’s (either conscious or not) understanding of what is beneficial or harmful for their health. These beliefs vary both within and between societies. Participatory approaches in policy-making processes may strengthen Social Circumstances in communities and have positive health effects [5].

### Social stratification process

The Social Stratification Process refers to the ways a society is hierarchically stratified, based on systematically unequal distribution of power, prestige, and resources, as well as discrimination [5]. These structural stratifiers interrelate with social relations assigning individuals to a Social Location. Social stratification indirectly determines differential health-related exposure, vulnerability, and consequences [5].

Power is intertwined in political, economic, and sociocultural relationships. It has either negative or positive denotation: for the former, it involves an imposition or domination (e.g., corporate lobbying against banning unhealthy food advertisement for children); for the latter, power is a human agency (e.g., empowering youth to voice their concerns about gun violence). Power is also presented at the micro- (e.g., households) and macro-level (e.g., governments). The reduction of health inequities requires macro-level changes to a more equal distribution of power.

Prestige refers to the status an individual holds in a social hierarchy, considering, for example, occupation, income and education [5]. Resources are tangible and intangible assets individuals have access to. Discrimination can be based on any aspect of Social Location, like age, sex, sexual identity, race/ethnicity, residence area, or income. The veiled or unveiled forms of discrimination limit one’s ability to access power, prestige, and resources. Likewise, power, prestige, and resources are interconnected with direct or indirect implications to inequities on physical, social, and mental health. As Social Stratification is a process, it has only indirect effects on health there is no direct indicator to measure its impact on health.

### Social location

Social Location, the product of Social Stratification, is the rank or position an individual is attributed to hold in a sociocultural and economic hierarchy within a society at a given time [5]. Interactions of multiple, intersecting social processes define one’s social position within the societal structure [15, 16]. This relational position is shaped by the interacting, intertwined influences of power relationships, access to resources, prestige, and discrimination.

Social Location can be measured through power-related measures (e.g., workplace control, household authority, gender roles), resources-based measures (e.g., income level and social class), prestige-based measures (e.g., educational achievement and occupational status), and discrimination (e.g., immigration status and religion) [15, 17]. It is worth highlighting some dimensions can be looked at from different perspectives. For instance, occupational status can be seen as a resource- and/or prestige-based measure. Gender (i.e., the socially constructed differences (designating masculinity and femininity roles) attributed to biological differences between sexes) can be a source of power but also discrimination with unfair treatment in societies with gender-based social hierarchies. While measuring one aspect of Social Location is methodologically possible, its findings are better interpreted using an intersectionality approach [15]. Recognising the combined, intersecting Social Locations an individual holds allows for a better understanding of the cumulative effects on health [18].

Social Location influences health indirectly. Those in lower Social Locations face an increased likelihood of being victimised or discriminated against and, hence, may be exposed to Psychological Stressors and experience stress [17, 19]. For instance, a new immigrant with limited official language proficiency may feel isolated in his workplace. Social Location is also linked to Social Circumstances, providing more or less social cohesion or social capital. Belonging to a Lesbian, Gay, Bisexual, and Transgender community may provide a non-heterosexual with human rights protection and social support to sexual orientation and gender identity acceptance.

Social Location may influence access to Material resources [19, 20]. The gender pay gap favouring men is an expression of discrimination against working women. The so-called motherhood penalty leads to childless woman and mothers having fewer chances for promotion and lower wages. The association between Social Location and Environment may determine where a person lives, works, studies, and plays. New immigrants tend to move in and frequent neighbourhoods with higher concentrations of people sharing the same ethnic and cultural identity. In addition to removing cultural and languages barriers and expanding migrant networks (an aspect of Social Circumstances), these ethnic enclaves concentrates a higher number of services and facilities (e.g., food outlets, religious places, social gatherings), which resembles the home country’s living conditions [21].

Health Beliefs are shaped by one’s social position in society. People sharing the same Social Location may also share a similar set of health-related values and ideologies. For example, traditions and beliefs of breast milk meeting nutritional needs of infants (e.g., colostrum perceived as bad milk) vary across ethnic groups leading to differences in the exclusive breastfeeding practises in the infant’s first 6 months of life [22, 23]. Social Location interacts with the Acceptability in healthcare system. Respectful service delivery deals with sensitivity to all Social Locations and includes the provision of services free from discrimination. Where healthcare is provided in a fashion unacceptable to certain Social Location groups, there may be less use the available Health-promoting Resources, even if the Need exists. For instance, a recent Canadian study showed growing healthcare inequities among Indigenous peoples are a result of systematic racism in clinical practises, leading to delay or discontinuity of seeking healthcare [24].

### Material circumstances

Material Circumstances refer to the financial means (income and material or intangible assets) allowing purchase and consumption [5] for ensuring healthy, dignifying living conditions. Material Circumstances include resource-based measures related to (a) satisfaction of basic needs (e.g., food security and house ownership); (b) possession of household amenities (e.g., central heating and washing machine); (c) potential consumption of social goods and services, including education and healthcare. The HEMF allows for exploring absolute income hypotheses and material deprivation mechanisms (individual’s own material circumstances) generating and reproducing health inequities [25, 26].

By directly influencing a number of SDOH, Material Circumstances have an indirect effect on Health State. Material Circumstances impact the Social Location an individual holds: lacking material resources may position an individual lower in the social ladder, leading to their marginalisation [27]. The scarcity of material resources or low income may act as a Psychosocial Stressor (either acute or chronic), causing stress and poor health [5, 26]. Material Circumstances also influence Environment, e.g. determining where a person can afford to live and the housing conditions [1, 28]. Material Circumstances, along with aspects of Social Location, shape Health-related Behaviours and Beliefs [1]. Depending on their access to material resources, individuals may share beliefs that socio-economically and culturally represent their group status. For instance, high income people are more likely to believe in the health benefits associated with regular medical check-ups than low income people. Lack of or insufficient material resources may affect people’s abilities to adopt and sustain good Health-related Behaviours, by limiting them, for instance, from engaging in sports and other leisure-time physical activities [1]. Material Circumstances can directly affect Health-related behaviours (e.g., the high cost of vegetables and fruits may lead low-income people to consume low-cost energy dense foods instead of nutritious foods [29, 30]).

Material Circumstances are connected with Appraisal and Coping mechanisms. The appraisal of challenges, particularly financial ones, may depend upon the material resources available to the individual. Material Circumstances can also substitute for social supports through the use of, for example, professional psychiatric services. Material Circumstances have a direct effect on Accessibility [31]: people with limited income and no health insurance may find more difficulty in accessing a medical laboratory for exams, reducing their likelihood of using Health-promoting Resources. Finally, Material Circumstances may mediate the relationship between Utilisation of Health-promoting Resources and Health Outcome [1]. For example, people lacking financial resources to continue using a medical facility for treatment may experience no improvement in their Health Outcome.

### Social circumstances

The HEMF incorporates the concepts of social cohesion at the population level and social capital at the individual level under the umbrella Social Circumstances. Despite its growing popularity in public health [1, 32], social capital has still no single, uncontested definition [5, 32–34], which has resulted in a plurality of measures used in health studies and mixed empirical results [33]. The HEMF uses the definitions developed by Carpiano [33]. Social cohesion refers to the patterns of social interactions and values emerging from these relationships, such as trust and norms or reciprocity [33]. Social cohesion is a social process through which social capital can be produced. Social capital focuses on the resources available to the individual in their social networks [33, 35]. The HEMF recognises social capital can be beneficial or harmful to health [36] (e.g., exclusion of outsiders or participation in ‘health-damaging’ networks [33, 34, 37]).

Social capital theories have evolved within public health literature, leading to a distinction of social capital forms: 1) structural and cognitive social capital; and 2) bonding, bridging, and linking. Structural social capital involves the extent and intensity of social connections or engagement in network activities, whereas cognitive social capital refers to perceptions of trust, solidarity, and reciprocity, and social support [38, 39].

Bonding capital refers to tangible or intangible resources that people with the similar social background can access through their participation in social networks [32]. It is characterised by strong ties based on trust and social support among people who consider themselves sharing the same social identity [40, 41]. Bridging social capital comprises potential resources that can be accessed by people with different social background [32]. These people who are loosely connected develop weaker ties, which are anchored in respect and mutuality [41]. Lastly, linking social capital is defined as vertical ties between people who are interacting across formal hierarchies of power and authority in society, such governments [40]. It is characterised by norms of respect and trust [41].

The HEMF shows Socioeconomic, Cultural, and Political Context shapes the type and strength of people’s interactions within social networks and the actual and potential resources derived from the networks [33, 36]. That aligns with an explanation of social capital in which some policies may lead an underinvestment or disinvestment in social infrastructure, and, consequently, in social capital [25, 36, 42].

Social Location may affect Social Circumstances [33, 35]. For instance, people with power, prestige, and access to resources may be able to convert them into social capital [35]. Social Location may also determine which social networks an individual might be able to participate, limiting their access to more or less resources emanating from the networks [33, 35].

Individuals who lack (or have insufficient) Material Circumstances may mobilise collective resources available in their social networks to obtain material and non-material resources (e.g., information, and emotional or instrumental support) and achieve their goals. In this case, Social Circumstances may moderate the relationship between Material Circumstances and Environment, Health-related Behaviours and Beliefs leading to a particular Health State [43]. Social Circumstances may moderate the association between Utilisation of Health-promoting Resources and Health Outcome [44, 45] or have a direct influence in Accessibility [39, 44]. Further, Social Circumstances may affect Appraisal and Coping skills [46], by providing resources to people deal with a stressful situation [19]. For instance, Social Capital may boost one’s Appraisal and Coping skills, helping mitigate the impact of poor Material Circumstances by buffering the negative effects of Psychosocial Stressors (e.g., job loss and death of a loved one). Finally, it is worth highlighting social cohesion may influence health, regardless of the social capital. For instance, feeling connected socially may impact one‘s happiness and quality of life [4].

### Biology

Biology includes biological factors (age, sex, genetics, and hormones) associated with susceptibility to certain diseases and injuries [47], which can result in changes in Health State. More broadly, embodiment theory suggests that exposures during critical periods and accumulation of exposures over the life course are reflected in biology [48]. For instance, the effects of ageing on musculoskeletal system lead to weakening of bone density and increased susceptibility to arthritis or osteoporosis among older adults.

### Environment

Environment is a broad category involving area-based measures and physical and social features of the space. Area-based measures can be at the aggregate or integral level. Aggregate measures refer to the composition of characteristics of people living in the same area (e.g., percentage of residents living below poverty line). Integral or global measures refer to contextual or group level constructs; i.e., characteristics that cannot be reduced to the group of individuals (e.g., population density) [49].

Environment also encompasses the concept of *place:* a combination of the physical characteristics and meanings attributed to the space where they live, work, or play. Place is also about the context per se, including the absence or presence of environmental tangible and intangible features, including natural (e.g., climate and geographic landmarks), built (e.g., sewage system and traffic calming measures), and sociocultural features of the environment (e.g., community’s reputation and graffiti) [28].

Aligned with socioecological models [50], the HEMF shows that Environment is influenced by Socioeconomic, Cultural, and Political Context, Material Circumstances, Social Location, and Social Circumstances. In turn, Environment influences Psychosocial Stressors [51]. An unsafe community (either defined objectively through crime statistics or subjectively through perceptions), gentrification processes (where socio-economically disadvantaged families can no longer afford renting costs due to high-income newcomers), or a work environment of high demand, low control jobs are examples of how Environment can be a source of chronic stress.

The Environment can also encourage people’s engagement in health-protecting or -damaging behaviours [5]. Consider how the built Environment influences people’s ability to actively commute to work when pedestrian and bicyclist infrastructure is available. Likewise, it can affect Health States through, for example, the positive effects of neighbourhood greenness on mental health [52]. Environment is also linked to Biology (through gene expression); for instance, exposures to carcinogens (e.g., silica dust in mining industry [53] or ultraviolet radiation among outdoor workers [54]) may result in cellular damage leading to lung or skin cancer, respectively. Finally, Environment affects Accessibility to health-promoting resources. An example is the lack of availability of specialised healthcare units in remote communities.

### Health-related Behaviours

Health-related Behaviours refers to any activity undertaken by people that influences directly or indirectly their health. Common examples include alcohol consumption, diet and eating practises, physical activity, smoking, drug use, and sexual behaviours. Research shows Health-related Behaviours lie on the causal pathway to Health States [55]. While many behaviours act in only one pathway (e.g., lack of condom use resulting in the increased likelihood of a sexually transmitted disease), others operate in competing pathways. For instance, higher alcohol consumption is associated with liver diseases, but its moderate and occasional intake may be used as a Coping mechanism to reduce Stress and mitigate its negative effects on well-being. The HEMF captures both types of pathways.

### Health beliefs

Health Beliefs refer to individual or collective perceptions of what influences health in a positive or negative way. Evidence shows Health Beliefs affect Health-related Behaviours and Need for health-promoting services [56]. Health Beliefs are a key component in the way people rationalise the benefits or harms of adopting and maintaining certain Health Behaviours, such as in waterpipe tobacco smoking. Beliefs surrounding the ability to change Health-related Behaviours (health locus of control) affect the likelihood of attempting and successfully adopting a health-promoting behaviour or quitting a health-damaging behaviour. For example, those with a higher locus of control are more likely to attempt smoking cessation and be successful [57]. The weighing of benefits and risks as determinants of Need for a service can be seen in immunisation. The benefits (e.g., perceived vaccine efficacy or protection of others at high risk) and the risks (e.g., the beliefs of onset of neurological illnesses or adverse reactions) are key components to the immunisation decision [58].

### Pre-existing health state

Pre-existing Health State refers to any previous health state that can change the likelihood of occurrence of the particular Health State under analysis. For instance, it is well-known that obesity may lead to diabetes onset. If diabetes is examined as the Health State, obesity should be considered a Pre-existing Health State and only the social determinants of diabetes would be taken into account. However, obesity could be separately analysed as the Health State of interest along with its social determinants. Combining the two analyses would provide an overall picture of the impact of SDOH on diabetes.

### Psychosocial stressors

Psychosocial Stressors refer to any social, environmental or external challenge that requires an individual to adapt to it [59]. These stressors can be acute (e.g., a recent life event such as job loss) or chronic (e.g., continuous daily discrimination based on sexual identity). Any of these may result in a Stress response.

Psychosocial Stressors originating from Social Location can be individual or structural in nature. The stressors from individual interactions may be experienced as discrimination and can be described by intersectionality theory [60], which recognises that stressors often become more complex and prevalent as the number of unique Social Locations increases [61]. For example, the stressors experienced by a black woman are more than just the sum of the stressors of being black plus the stressors of being a woman. Social exclusion is due to structural impediments indirectly created by the Socioeconomic, Cultural, and Political Context that discriminate and do not fully accommodate certain Social Locations [61]. For example, the lack of policies on accessible routes may exclude the physically disabled from fully participating in society.

### Appraisal and coping

Before a Psychosocial Stressor causes a Stress Response, there is a mediating process of Appraisal and Coping [62]. Appraisal refers to the evaluation of the event. Different social groups and individuals can appraise the same event differently. The Coping process is about a variety of potential strategies to deal with the Stressor.

Social Circumstances [61, 63], Material Circumstances and Health-Related Behaviours determine the suite of Coping strategies available to an individual. Material Circumstances and Social Circumstances represent resources that individuals facing a Stressor can draw upon to help cope, while Health-related Behaviours may express through or be intensified as coping mechanisms [5, 47]. The effectiveness of these Coping mechanisms combined with the Appraisal of the Psychosocial Stressor determines the level of stress experienced in the Stress Response.

### Stress response

The Stress Response can result in Biological changes. A Stress Response acts on multiple body systems (neuroendocrine, autonomic nervous, metabolic, and immune systems) and can change a wide variety of body markers [64]. These effects accumulate and can lead to any number of Health States including stroke, infectious diseases, diabetes, and shorter life expectancy [65]. For instance, a Stress Response may lead to a reduction of immune response and increase of hormone levels, affecting physical and mental health. Additionally, the Stress Response influences Health-related Behaviours. For example, a teenager experiencing a high level of stress may smoke cannabis as a method of dealing with the Stressors. Substance abuse may also be initiated by a Stress Response. Additionally, the adoption of certain Health-Related Behaviours may be a response to experienced stress determined by the link between Material Circumstances and Psychosocial Stressors [5, 47].

### Health state

Health State consists of any health description and/or measurement of an individual at a given time [47] and may involve any aspect of physical or mental health and well-being [5]. Health States are influenced by Biology (e.g., estrogen fluctuations leading to higher prevalence of depression among women), Environment (e.g., no access to clean water in the neighbourhood increasing likelihood to infectious diseases), Health-related Behaviours (e.g., poor dental hygiene may cause gum diseases), Pre-existing Health States (e.g., uncontrolled diabetes may trigger retinopathy) and Stress Response (raised cortisol output leading to metabolic syndrome). In turn, Health States may affect the Need for seeking healthcare. For example, a teacher of a student with suicidal thoughts and behaviours may identify a need to get professional help for the child.

### Need

Need refers to either self-perceived or professionally evaluated Need to utilise Health-promoting Resources. Need is determined by the Health State, Health Beliefs, and Continuity of Care. Health-related concerns, symptoms of illnesses or diseases, injuries or disabilities can result in a perceived or professionally evaluated Need for care [9]. Health Beliefs impact perceived Need by representing the values and health knowledge used to decide when to seek care for a health issue. In the realm of vaccines, certain groups who do not believe in its benefits may not seek immunisation, despite a medically evaluated Need. Continuity of Care can directly influence professionally evaluated Need. Regular contact with healthcare provider can identify opportunities for screening and early diagnoses, all leading to a professionally evaluated or self-perceived Need for care.

### Health policy context

The health system is a SDOH mitigating differences in exposure and vulnerability to health conditions through the provision of physically accessible, affordable, timely, and effective healthcare [5, 66, 67]. Explicitly developed in the HEMF, the Health Policy Context - itself shaped by the broader Social, Economic, and Political Context - is the nexus of policies and decisions influencing Availability of health-promoting resources and a number of dimensions of health system quality, including Acceptability, Appropriateness, Safety, Effectiveness, and Continuity.

Availability of health-promoting resources is defined within the Health Policy Context. Policies, programs, and resources allocation affect the presence, location, and organisation of healthcare physical infrastructure, the provision of health supplies and services, staffing, and human resource management. The Health Policy Context may determine processes that inform practises for healthcare delivery and its quality. Protocols and guidelines designed in the Health Policy Context may ensure: 1) services are respectful and responsive to individuals’ needs and preferences (Acceptability); 2) there is a balance of evidence-informed health practises with individuals’ needs and preferences for appropriate healthcare (Appropriateness); 3) care delivery prevents and minimise health risks (Safety); 4) use of best and updated scientific knowledge and practises for optimal health outcome for the individual (Effectiveness). Finally, it also influences Continuity of care, ensuring health services are well coordinated and integrated.

### Availability of health-promoting resources

Influenced by Health Policy Context, Availability of Health-promoting Resources represents the infrastructure and its corresponding organisation for healthcare provision. It captures 1) the presence of health professionals, services, and supplies; 2) the existence and spatial location of physical infrastructure (e.g., facilities and ambulances); and 3) the health system’s organisational characteristics, including waiting times and hours of operation. Similar to a shortage of healthcare workers, the organisational characteristics of the health system may restrict the Availability of Health-promoting Resources, negatively affecting its use. As seen in the HEMF, Availability modifies the relationship between Need and Utilisation of Health-promoting Resources.

### Acceptability, appropriateness, safety, effectiveness, and continuity

The Health Policy Context influences the quality of health-promoting resources through Acceptability, Appropriateness, Safety, Effectiveness, and Continuity. Acceptability is the respectfulness and responsiveness of healthcare to individual’s needs, preferences, and expectations [8]. Policies and programs influence the level of Acceptability in terms of the provision of respectful and responsive services regardless of individual’s Social Location, creating a healthcare setting free of discrimination. Appropriate care is receiving a suitable, evidence-based health service that is balanced with individual needs and preferences [8]. The Health Policy Context can encourage Appropriate care through the normalisation of evidence-based practice. The Health Policy Context also affects provider behaviours through incentive structures, for example, different payment schemes. Both Acceptability and Appropriateness interact with Need to determine Utilisation of Health-promoting Resources.

Safety refers to mitigation of risks when an individual is receiving care in the health system [8]. Effectiveness considers the use of current scientific knowledge and best practises to achieve optimal health outcomes for the individual [8]. Safety and Effectiveness are effect modifiers of the Utilisation of Health-promoting Resources and Health Outcome relationship.

Continuity of Care describes how the healthcare provided to an individual is delivered over time. It ensures that the health services are well coordinated and integrated for predictable and coherent delivery of care [68, 69]. Continuity of Care affects Utilisation of Health-promoting Resources after a Need is identified. This can be seen in the case of an individual with a chronic disease, where provider continuity has been associated with higher medication adherence [70]. It can also modify the effect of Utilisation of Health-promoting Resources on Health Outcome.

### Accessibility

Accessibility refers to the individuals’ ability to be able to use a health-promoting resource, once a Need is identified. Accessibility is directly influenced by Material Circumstances [27], Social Circumstances [39], and Environment [28]. Using transportation as an example, an individual without sufficient material resources may not have a car, creating a barrier to accessing a health-promoting resource. However, living in an Environment with high quality public transportation or having a positive social capital that allows them to obtain a ride can compensate for this material lack and provide access to the health-promoting resources.

### Utilisation of health-promoting resources

The HEMF captures Utilisation (or lack thereof) of Health-promoting Resources from the public, private, and non-for-profit sectors. These sectors provide primary, secondary, and tertiary healthcare services for prevention, diagnosis, and treatment of illnesses, diseases, injuries, and disabilities. Utilisation of Health-promoting Resources has a direct impact on the Health Outcome; e.g., engagement in physiotherapy treatment for sport injuries leading to full recovery. Utilisation is determined by Need with Availability, Accessibility, Acceptability, Appropriateness, and Continuity moderating the relationship.

### Health outcome

Health Outcome refers to a health state after any type of (potential) Utilisation of Health-promoting Resources. While the Utilisation of Health-promoting Resources directly influences the Health Outcome, a number of indirect factors play a role including Effectiveness of the health-promoting resource, Safety, Continuity of Care, Social Circumstances, and Material Circumstances. This Health Outcome can be professionally evaluated (e.g. successful removal of cancer) or perceived (a patient reported outcomes measure (PROM), e.g. using quality of life instruments).

## Discussion

The HEMF provides a synthesis of the effects of social determinants of both health and health service utilisation on health equity. Such a framework for measuring health equity comes with limitations and strengths. One limitation is that feedback loops are not illustrated in the diagram. However, they can be considered in data analysis; e.g., examining the impact of Health State on Material Circumstances or the influence of Pre-existing Health State on Social Location (Additional file 2). The succinctness in the HEMF could be considered another limitation. For example, some obesity frameworks are quite detailed and complex (see [71]), whereas not all specific elements are explicitly shown in the HEMF. However, for any specific health dimension under examination, it is possible to unpack some of the concepts presented to obtain the level of detail required. As such, the HEMF provides a useful general framework for measuring health equity.

The HEMF has two main strengths. First, HEMF provides a more comprehensive model to study health equity because it is built upon on and integrates current research and frameworks on SDOH and health system utilisation. It simultaneously combines multiple SDOH that may not be present in any particular framework.

Second, by operationalising existing frameworks, the HEMF provides overarching direction to empirical work (statistical modelling and performance measurement), as well as identifying intervention points for strategic public policies. Each of HEMF mechanisms can be developed into indices measuring the impact of SDOH on individual or population health. From a surveillance perspective, the HEMF helps with subpopulation comparisons and monitoring of changes over time for the design or evaluation of programs and policies. It supports the development of evidence-based interventions that are likely to have the largest, long-lasting impact on health equity. Considering its breadth, the HEMF may also be relevant and useful for other public sectors (e.g., labour, social services, and education) in their understanding of how their policies promote population health and health equity. The measurement results also help identify opportunities for intersectoral, collaborative plans of action to improve health equity.

## Conclusions

This paper presented a complex, overarching measurement framework for health equity. The HEMF is a synthesis of existing SDOH and health system utilisation frameworks and current literature. Yet, its purpose extends to focus on measurement. It is specifically designed to help identify and measure the interrelationships between political and socio-cultural context, health system-related policies and programs, material and social circumstances, environment, biological and psychosocial factors, perceived and evaluated needs, social location, health-related behaviours, beliefs, and health state and outcomes. It provides guidance to the design of research on public health and health services, application of statistical methods for academic studies or surveillance systems, and development of policies and programs to promote health equity. More specifically, it clearly delineates the causal pathways allowing for effective statistical modelling and development of evidence of the effects of SDOH on health equity. It also highlights policy entry points, both for health and related organisations whose purview have an effect on health. In summary, the Health Equity Measurement Framework (HEMF) provides a measurement framework informing actions that target health inequities.

## Additional files

Additional file 1: Key Perspectives and Concepts in Developing the HEMF. The additional file identifies the frameworks and concepts that provided the initial foundations for the HEMF. The reasons and perspectives behind their use are noted. (PPTX 48 kb)

Additional file 2: Example Extending the HEMF to Include Feedback Loops. The figure illustrates a portion of the HEMF focusing on Diabetes and Obesity with examples of feedback loops. (PPTX 62 kb)

## Abbreviations

HEMF: Health Equity Measurement Framework
SDOH: Social Determinants of Health
