# Correction to: The health equity measurement framework: a comprehensive model to measure social inequities in health

**DOI:** 10.1186/s12939-019-0949-7

**Published:** 2019-04-23

**Authors:** Douglas C. Dover, Ana Paula Belon

**Affiliations:** 1 Alberta Health, Government of Alberta, Edmonton, AB Canada; 2 Concordia University of Edmonton, Edmonton, AB Canada; 3 School of Public Health, University of Alberta, Edmonton, AB Canada

**Correction to: International Journal for Equity in Health (2019) 18:36**

**https://doi.org/10.1186/s12939-019-0935-0**

Following publication of the original article [1], the author reported a mistake in the caption related to Fig. 2.

**Fig. 2:**
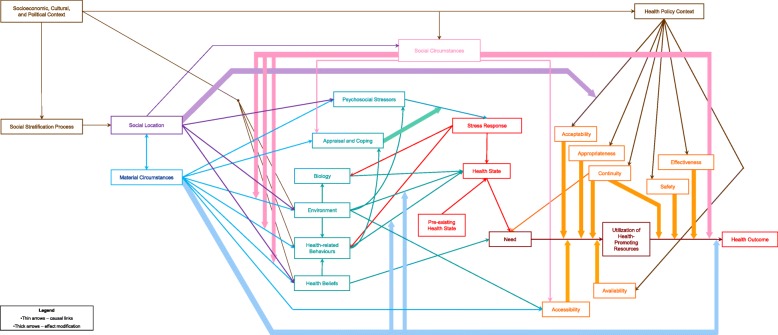
Health Equity Measurement Framework

The published caption “The blue line between “Social Location” and “Material Circumstances” should be double headed, having an arrow head at both ends” needs to be replaced with “Health Equity Measurement Framework”.

Also, in Fig. 2 the arrow linking “Social Location” and “Material Circumstances” should be pointing at both directions.

The original article has been corrected as well.

The publisher apologizes for any inconvenience caused by this error.

## References

[1] Dover DC, Belon AP (2019). The health equity measurement framework: a comprehensive model to measure social inequities in health. Int J Equity Health.

