# Supplementary material for: The health equity measurement framework: a comprehensive model to measure social inequities in health
Source: Int J Equity Health. 2019 Feb 19;18:36. doi: 10.1186/s12939-019-0935-0 (PMC6379929; doi:10.1186/s12939-019-0935-0)
Supplement: Supplementary file 1 — Key Perspectives and Concepts in Developing the HEMF. The additional file identifies the frameworks and concepts that provided the initial foundations for the HEMF. The reasons and perspectives behind their use are noted. (PPTX 48 kb) [file 12939_2019_935_MOESM1_ESM.pptx]

## Slide 1
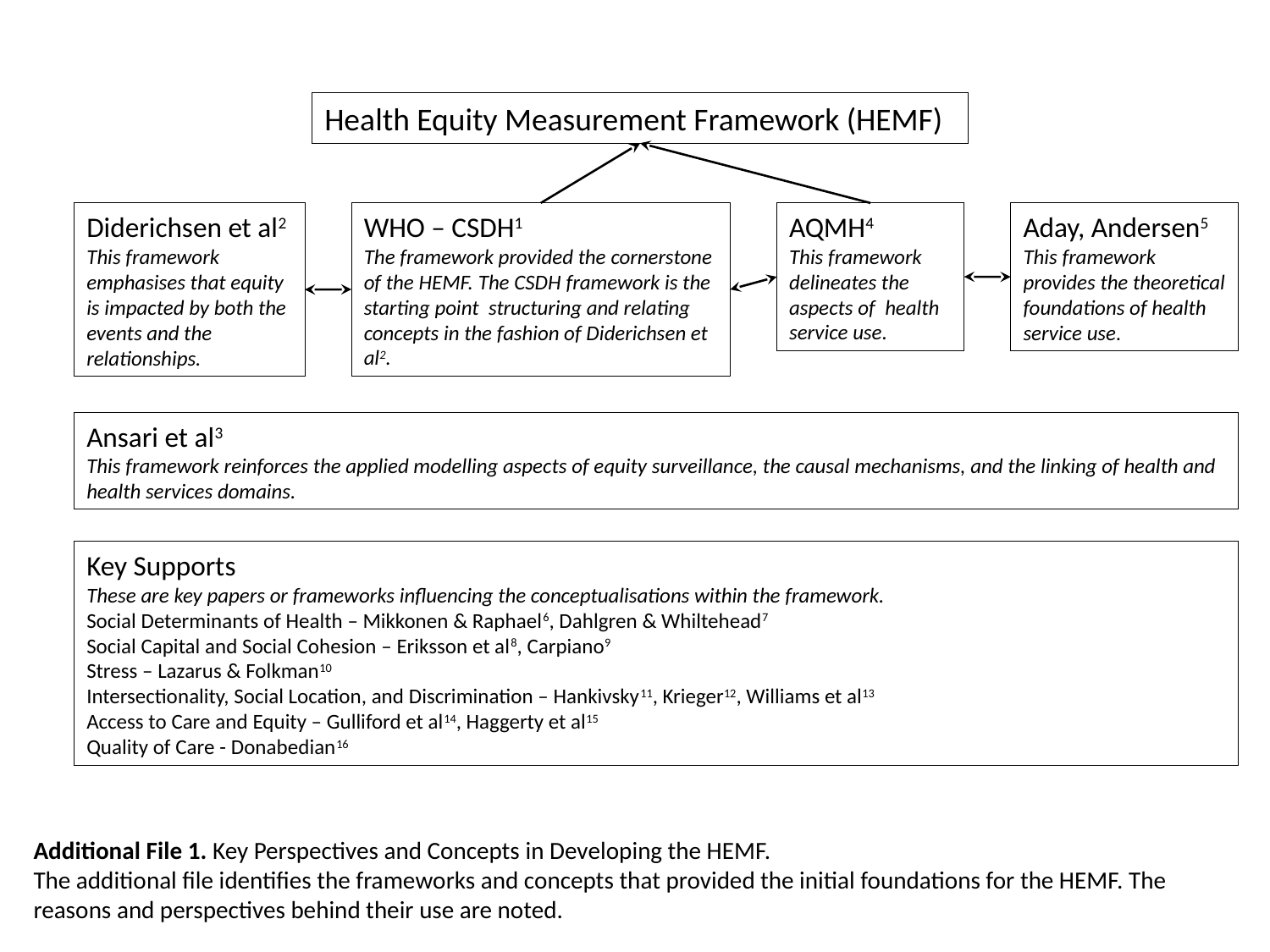

Health Equity Measurement Framework (HEMF)
WHO – CSDH1
The framework provided the cornerstone of the HEMF. The CSDH framework is the starting point structuring and relating concepts in the fashion of Diderichsen et al2.
AQMH4
This framework delineates the aspects of health service use.
Diderichsen et al2
This framework emphasises that equity is impacted by both the events and the relationships.
Aday, Andersen5
This framework provides the theoretical foundations of health service use.
Ansari et al3
This framework reinforces the applied modelling aspects of equity surveillance, the causal mechanisms, and the linking of health and health services domains.
Key Supports
These are key papers or frameworks influencing the conceptualisations within the framework.
Social Determinants of Health – Mikkonen & Raphael6, Dahlgren & Whiltehead7
Social Capital and Social Cohesion – Eriksson et al8, Carpiano9
Stress – Lazarus & Folkman10
Intersectionality, Social Location, and Discrimination – Hankivsky11, Krieger12, Williams et al13
Access to Care and Equity – Gulliford et al14, Haggerty et al15
Quality of Care - Donabedian16
Additional File 1. Key Perspectives and Concepts in Developing the HEMF.
The additional file identifies the frameworks and concepts that provided the initial foundations for the HEMF. The reasons and perspectives behind their use are noted.

## Slide 2
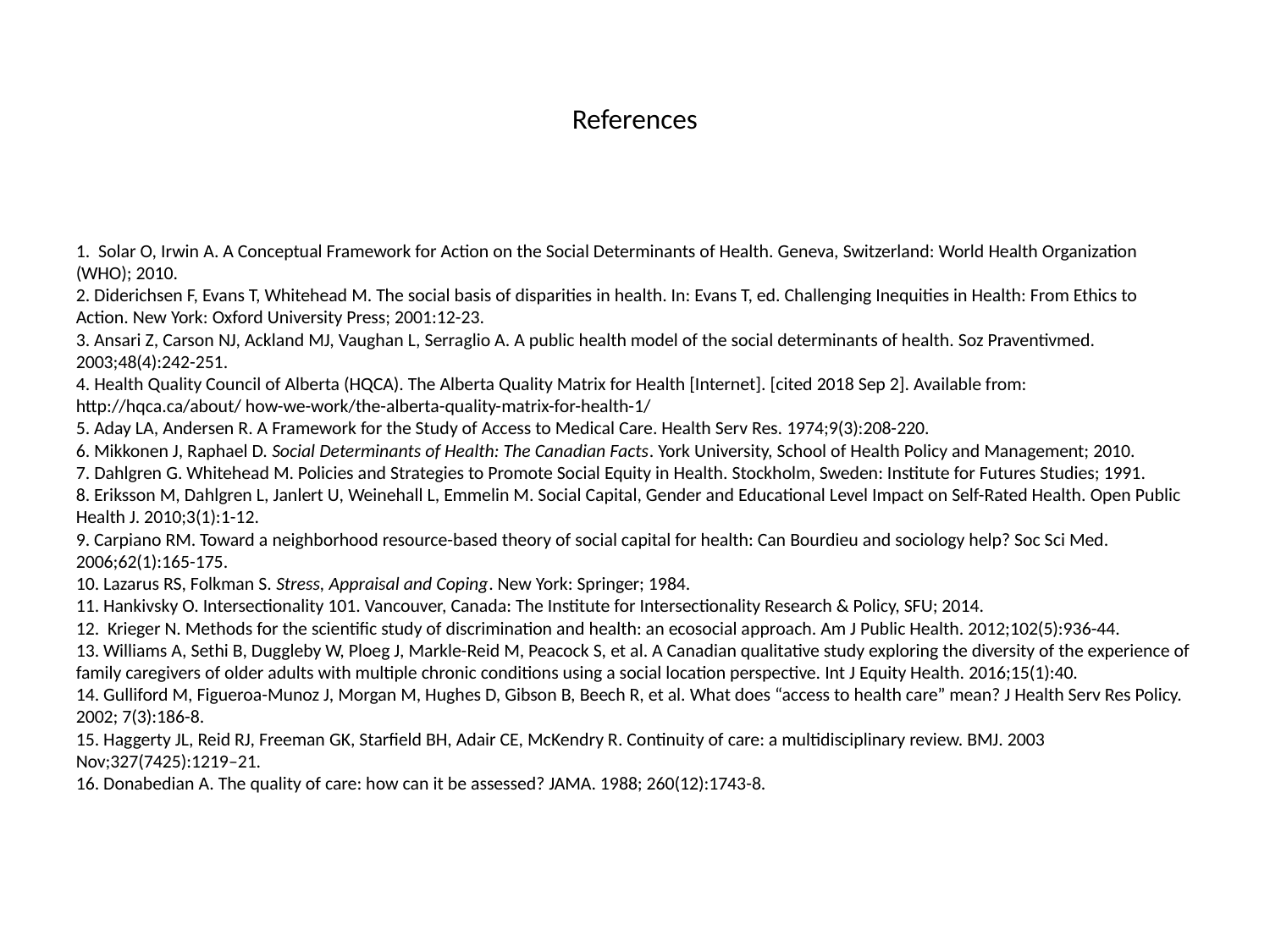

# References
1. Solar O, Irwin A. A Conceptual Framework for Action on the Social Determinants of Health. Geneva, Switzerland: World Health Organization (WHO); 2010.
2. Diderichsen F, Evans T, Whitehead M. The social basis of disparities in health. In: Evans T, ed. Challenging Inequities in Health: From Ethics to Action. New York: Oxford University Press; 2001:12-23.
3. Ansari Z, Carson NJ, Ackland MJ, Vaughan L, Serraglio A. A public health model of the social determinants of health. Soz Praventivmed. 2003;48(4):242-251.
4. Health Quality Council of Alberta (HQCA). The Alberta Quality Matrix for Health [Internet]. [cited 2018 Sep 2]. Available from: http://hqca.ca/about/ how-we-work/the-alberta-quality-matrix-for-health-1/
5. Aday LA, Andersen R. A Framework for the Study of Access to Medical Care. Health Serv Res. 1974;9(3):208-220.
6. Mikkonen J, Raphael D. Social Determinants of Health: The Canadian Facts. York University, School of Health Policy and Management; 2010.
7. Dahlgren G. Whitehead M. Policies and Strategies to Promote Social Equity in Health. Stockholm, Sweden: Institute for Futures Studies; 1991.
8. Eriksson M, Dahlgren L, Janlert U, Weinehall L, Emmelin M. Social Capital, Gender and Educational Level Impact on Self-Rated Health. Open Public Health J. 2010;3(1):1-12.
9. Carpiano RM. Toward a neighborhood resource-based theory of social capital for health: Can Bourdieu and sociology help? Soc Sci Med. 2006;62(1):165-175.
10. Lazarus RS, Folkman S. Stress, Appraisal and Coping. New York: Springer; 1984.
11. Hankivsky O. Intersectionality 101. Vancouver, Canada: The Institute for Intersectionality Research & Policy, SFU; 2014.
12.  Krieger N. Methods for the scientific study of discrimination and health: an ecosocial approach. Am J Public Health. 2012;102(5):936-44.
13. Williams A, Sethi B, Duggleby W, Ploeg J, Markle-Reid M, Peacock S, et al. A Canadian qualitative study exploring the diversity of the experience of family caregivers of older adults with multiple chronic conditions using a social location perspective. Int J Equity Health. 2016;15(1):40.
14. Gulliford M, Figueroa-Munoz J, Morgan M, Hughes D, Gibson B, Beech R, et al. What does “access to health care” mean? J Health Serv Res Policy. 2002; 7(3):186-8.
15. Haggerty JL, Reid RJ, Freeman GK, Starfield BH, Adair CE, McKendry R. Continuity of care: a multidisciplinary review. BMJ. 2003 Nov;327(7425):1219–21.
16. Donabedian A. The quality of care: how can it be assessed? JAMA. 1988; 260(12):1743-8.
