# Supplementary material for: The health equity measurement framework: a comprehensive model to measure social inequities in health
Source: Int J Equity Health. 2019 Feb 19;18:36. doi: 10.1186/s12939-019-0935-0 (PMC6379929; doi:10.1186/s12939-019-0935-0)
Supplement: Supplementary file 2 — Example Extending the HEMF to Include Feedback Loops. The figure illustrates a portion of the HEMF focusing on Diabetes and Obesity with examples of feedback loops. (PPTX 62 kb) [file 12939_2019_935_MOESM2_ESM.pptx]

## Slide 1
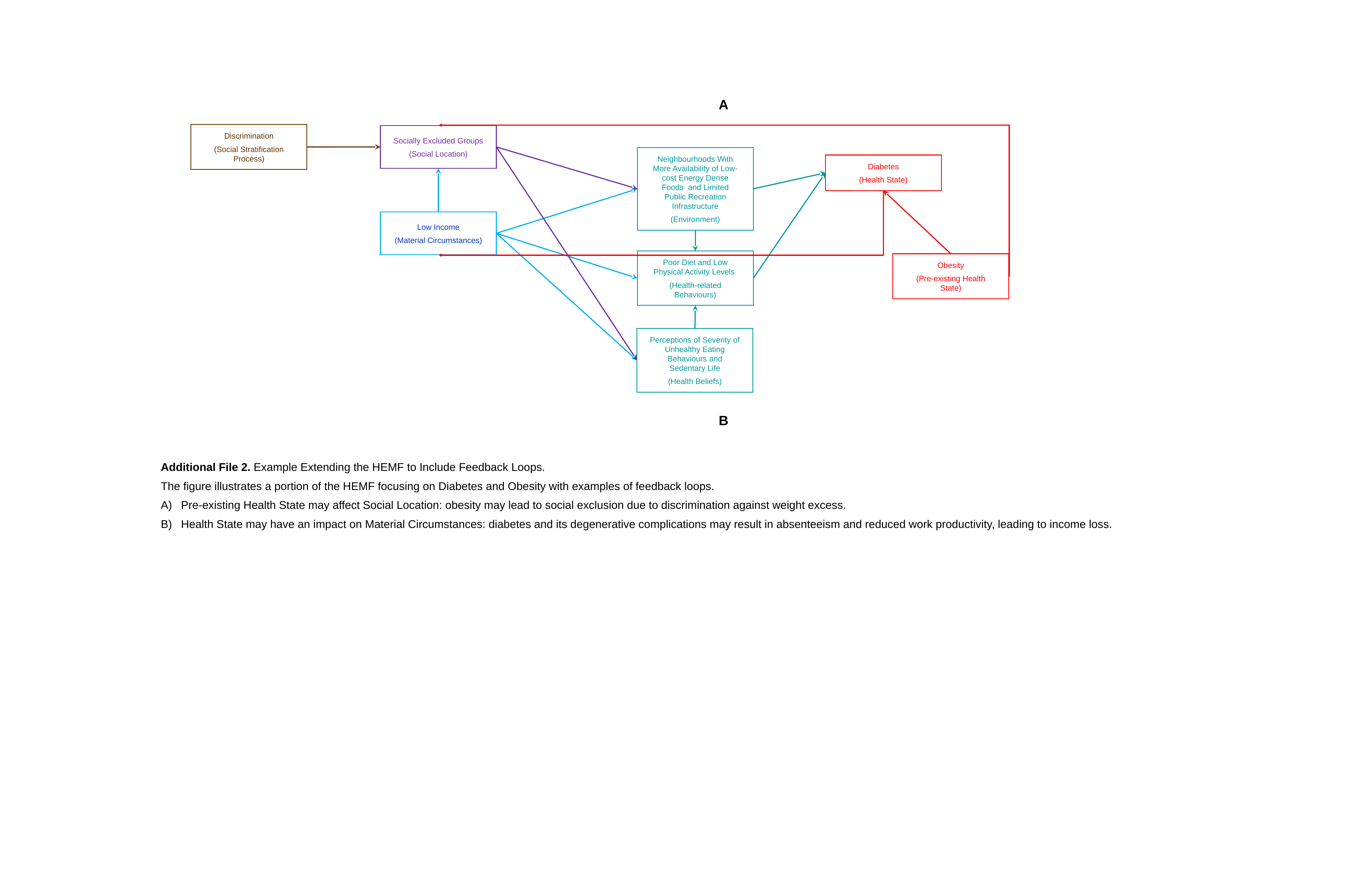

A
Socially Excluded Groups
(Social Location)
Discrimination
(Social Stratification Process)
Diabetes
(Health State)
Neighbourhoods With More Availability of Low-cost Energy Dense Foods and Limited Public Recreation Infrastructure
(Environment)
Low Income
(Material Circumstances)
Poor Diet and Low Physical Activity Levels
(Health-related Behaviours)
Obesity
(Pre-existing Health State)
Perceptions of Severity of Unhealthy Eating Behaviours and Sedentary Life
(Health Beliefs)
B
Additional File 2. Example Extending the HEMF to Include Feedback Loops.
The figure illustrates a portion of the HEMF focusing on Diabetes and Obesity with examples of feedback loops.
Pre-existing Health State may affect Social Location: obesity may lead to social exclusion due to discrimination against weight excess.
Health State may have an impact on Material Circumstances: diabetes and its degenerative complications may result in absenteeism and reduced work productivity, leading to income loss.
